# Reversal of neurobehavioral social deficits in dystrophic mice using inhibitors of phosphodiesterases PDE5A and PDE9A

**DOI:** 10.1038/tp.2016.174

**Published:** 2016-09-27

**Authors:** M S Alexander, M J Gasperini, P T Tsai, D E Gibbs, J M Spinazzola, J L Marshall, M J Feyder, M T Pletcher, E L P Chekler, C A Morris, M Sahin, J F Harms, C J Schmidt, R J Kleiman, L M Kunkel

**Affiliations:** 1 Division of Genetics and Genomics, Boston Children's Hospital, Boston, MA, USA; 2 Departments of Pediatrics and Genetics, Harvard Medical School, Boston, MA, USA; 3 The Stem Cell Program, Boston Children's Hospital, Boston, MA, USA; 4 The F.M. Kirby Neurobiology Center, Translational Neuroscience Center, Department of Neurology, Boston Children's Hospital, Harvard Medical School, Boston, MA, USA; 5 Rare Disease Research Unit, Pfizer, Cambridge, MA, USA; 6 Neuroscience Research Unit, Pfizer Global Research and Development, Cambridge, MA, USA; 7 The Manton Center for Orphan Diseases, Boston, MA, USA; 8 Harvard Stem Cell Institute, Cambridge, MA, USA

## Abstract

Duchenne muscular dystrophy is caused by mutations in the *DYSTROPHIN* gene. Although primarily associated with muscle wasting, a significant portion of patients (approximately 25%) are also diagnosed with autism spectrum disorder. We describe social behavioral deficits in dystrophin-deficient mice and present evidence of cerebellar deficits in cGMP production. We demonstrate therapeutic potential for selective inhibitors of the cGMP-specific PDE5A and PDE9A enzymes to restore social behaviors in dystrophin-deficient mice.

## Introduction

Duchenne muscular dystrophy (DMD) affects approximately 1:5000 live male births, and is caused by pathogenic mutations in the *DYSTROPHIN* gene.^1, 2^ DMD patients experience skeletal muscle degeneration, particularly in the diaphragm muscles, and severe cardiac arrhythmias that often lead to death in early adulthood. Consistent with the clinical range of symptoms of DMD, dystrophin is highly expressed in skeletal muscle, cardiac muscle and restricted regions of the brain including the cerebellar Purkinje and cortical neurons.^3, 4^ A significant number of DMD patients also have significantly reduced intelligent quotient scores, deficits in responses to bright-light stimuli and electroretinogram measurements,^5, 6, 7, 8, 9^ and meet the diagnostic criteria for autism spectrum disorder (ASD).^6, 10, 11, 12^ Historical characterization of DMD by Guillaume Duchenne included reports of significant mental deficits in subsets of patients afflicted with the disorder.^13^ Further study of DMD boys carried out in the 1970's found a significant portion of the affected population performed poorly on school exams, had difficulty reading, exhibited emotional disturbances and overall decreased verbal communication skills^14^ consistent with classical studies that characterized lower intelligence scores from DMD boys compared with their aged-match peer cohorts.^15^ Historical studies of DMD diagnosed this affected population of boys solely under the classification of intellectual disability,^16^ however, standardized refinement of the ASD diagnosis via the Diagnostic and Statistical Manual of Mental Disorders guidelines^17^ has contributed to a greater appreciation for the specificity of many cognitive deficits within social domains^18^ ^19^ now ascribed to the autism spectrum. Recent work has shown that DMD boys had significant immediate memory and reading comprehension deficits that translated to lower academic achievement and lower test scores when compared with their peer groups.^20, 21, 22^ In most rigorous comparisons, DMD boys score at least one full standard deviation below the mean Full Scale Intelligent Quotient (score of 80) of aged-match peer cohorts (score of 100).^23^ Boys with the milder Becker muscular dystrophy (BMD) produce variable amounts of a partially functional, truncated dystrophin protein, and some develop significant learning difficulties and behavioral problems, albeit with a much less homogeneous cognitive phenotype, consistent with variability in the amounts of dystrophin protein produced.^24^

Modern diagnostic criteria for defining the ASD scale have revealed a significant fraction (20–30%) of DMD boys exhibit comorbid ASD neurobehavioral deficits.^10^ Significantly, when evaluating children with ASDs for commonly associated comorbid disorders, the generalized category of 'muscular dystrophies' is the second most comorbid set of diagnoses with children diagnosed with ASDs.^25^ These results strongly support the hypothesis that the dystrophin protein in the brain is essential for normal cognitive function in humans.^26^ The dystrophin protein and the dystrophin-associated protein complex has been detected at the synapses of the hippocampus and regions of the cerebellum of dystrophin-deficient mice^3, 4, 27, 28^ and patients,^29^ where it is hypothesized to regulate the postsynaptic anchoring of GABA receptors required for normal inhibitory synaptic transmission.^30^ Evidence of decreased postsynaptic GABA receptors on Purkinje cells of dystrophin-deficient mice have been observed using whole-cell patch clamp recordings.^31^ Loss of the Dp140 dystrophin protein isoform owing to pathogenic DMD mutations has been implicated in the correlation between patients with DMD and cognitive impairment.^26, 32, 33^ Nevertheless, a direct link between the loss of expression of the Dp140 dystrophin protein isoform as the direct cause of ASD in DMD patients has not been fully established.

Most pharmacological treatments for DMD focus on blocking the associated inflammation and maintaining muscle strength via the use of glucocorticoids,^34^ with no attention paid to potential treatments that may improve cognitive or autism-related symptoms. Our lab has previously demonstrated that chronic treatment with the selective PDE5A inhibitor sildenafil can improve phenotypes of muscle structure, performance and overall lifespan in the dystrophin-deficient (*sapje*) mutant zebrafish.^35^ Parallel studies in the *mdx* (dystrophin exon 23 mutation) mutant mice treated with sildenafil resulted in dystrophic mice with reduced dystrophic symptoms of diaphragm muscle weakness and reduced overall muscle fibrosis.^36^ Similarly, *mdx* mice given the longer-acting tadalafil (Cialis) had a switch towards slower, more oxidative muscle fibers, alteration of lipid metabolism and an overall amelioration of dystrophin-deficient clinical signs.^37^ A recent study in DMD boys given either sildenafil or tadalafil demonstrated that DMD patients had reduced muscle ischemia compared with untreated patients.^38^ These findings are consistent with an earlier clinical study in patients with BMD who showed improvements in a reduction of muscle ischemia via a NO-cGMP-dependent mechanism.^39^

Very little is known about the functional role of dystrophin in the brain, however, animal models of DMD exhibit a wide range of Purkinje cell deficits in excitability and plasticity, consistent with the high expression of dystrophin in this cell population.^4^ Dystrophin is highly expressed in cerebellar Purkinje neurons in addition to being expressed in other areas of the central and peripheral nervous systems^3^ (Supplementary Figure 1). Several compelling lines of evidence now suggest that neurodevelopmental cerebellar dysfunction contributes to social deficits in ASD.^40, 41, 42^ Cerebellar circuitry is highly conserved across mammals and plasticity in this circuitry is critically dependent upon cGMP-dependent postsynaptic long-term depression in Purkinje neurons, which is enhanced by cGMP PDE inhibitors.^43^ Furthermore, the PDE5A and PDE9A enzymes are highly enriched in cerebellar Purkinje neurons.^44, 45^ We hypothesized that dystrophin-deficient mouse models with reported evidence of Purkinje cell dysfunction will exhibit behavioral symptoms of impaired social approach behavior that are often associated with defects in the development of cerebellar circuitry. We further hypothesized that inhibitors of cGMP phosphodiesterases PDE5A and PDE9A will increase cerebellar cGMP and provide a therapeutic benefit for social approach behavior in dystrophin-deficient mouse models.

## Materials and methods

### Mice

Wild-type (C57BL/6 J; stock number 000664) and *mdx*^*5cv*^ (stock number 002379) mice were originally obtained from the Jackson Laboratory (Bar Harbor, ME, USA) and housed under sterile, pathogen-free conditions. Additional *mdx* mice (stock number 001801) and wild-type control C57BL10/ScSn (stock number 000476) mice were also purchased from the Jackson Laboratory used to obtain the cGMP data. All animal studies were approved by the Boston Children's Hospital Animal Resources at Children's Hospital (ARCH) Review Board (protocol number 12-10-2287 R).

### PDE compound preparation and administration

PDE5A inhibitor (sildenafil citrate/Viagra) and PDE9A inhibitor (PF-04749982; clinical candidate 19)^46^ were obtained from Pfizer as a powdered compound. The compound was reconstituted in mouse drinking acidified water (pH=3.0) and prepared fresh for each week of treatment. Sildenafil was administered to wild-type or *mdx*^*5cv*^ mice at 400 mg ml^−1^ via *ad libitum* inclusion in the drinking water, and the PDE9A inhibitor was included at 200 mg l^−1^ starting at 4 weeks after birth for eight continuous weeks adapted from a previously described dosing regimen.^36, 47^ The mice were evaluated for neurobehavioral performance at the end of the experiment at 12 weeks of age.

### Social approach

For non-drug-treated cohorts, the mice (adult males, 5 and 8 weeks old) were placed in the automated three-chamber assay system (Ethiovision XT software; Noldus, Leesburg, VA, USA). This experiment determines whether the test mouse shows a preference to a novel mouse or novel object (these are in chambers 1 and 3 and are switched from each chamber between each trial, and are enclosed in a see-through plastic cage with bars). This assay has been validated by several mouse models (Tsc1/2 Purkinje cell KO, BTBR, others) and the protocol has been described in detail.^48, 49, 50^ During the first trial, the test mouse (*mdx*^*5cv*^ or wild-type age-matched control male mice), is placed in the center chamber (referred to as chamber 2) in which two plastic barrier doors block access to the other two chambers (nothing is placed in chambers 1 or 2 in the initialization phase). The barriers are removed at the same time, and the mouse is monitored for a 10 min period in his movement between the three chambers. Afterwards, the mouse is placed in chamber 2, whereas the novel mouse or object (a round piece of PVC pipe) are placed either in chamber 1 or 3. The test mouse undergoes the trial in which the doors are removed, and the mouse will chose between chambers 1 or 3 (movement in the center chamber is also detected). The mouse is monitored over a 10 min interval, and its total movement, velocity, time spent in close interaction with the novel mouse or object, and other movement factors are measured. The experiment is conducted with 10 mice per genotype cohort to gain significant numbers. We quantified the amount of time each test mouse spent interacting with a novel object or a novel mouse. The experimenter was double-blinded to treatment group and genotype of the mice in each cage cohort.^48^

### Social novelty

We performed the social novelty three-chamber experiment similar to the social approach experiment and as previously described.^48, 50^ The test mouse was placed in the middle chamber (chamber 2) with barriers to the other chambers (chamber 1, familiar mouse) or the novel mouse (chamber 3, novel mouse). The familiar mouse was a littermate mouse, whereas the novel mouse was an age-, gender- and strain-matched mouse. The mouse is monitored over a 10 min interval, and its total movement, velocity, time spent in close interaction with the novel mouse or object, and other movement factors are measured. The experiment is conducted with 10 mice used per genotype cohort. We quantified the amount of time each test mouse spent interacting with a familiar mouse or a novel mouse. The experimenter was double-blinded to treatment group and genotype of the mice in each cage cohort following a previously described protocol.^48^

### cGMP measures

Male *mdx* mice and age-matched wild-type (C57BL10/ScSn) ranging from 6 to 7 weeks of age were ordered from Jackson Laboratories and acclimated for 1 week before the study (*N*=8 animals per group). Brain tissue measures of cGMP accumulation following drug administration were carried out as previously described.^51^ WT (C57BL10/ScSn strain), *mdx* (C57BL10/ScSn), WT (C57BL/6 J) and *mdx*^*5cv*^ (C57BL/6 J) mice were killed by focused microwave irradiation of the brain. The regions of interest were isolated and homogenized in 0.5 n HCl followed by centrifugation. The supernatant concentrations of cyclic nucleotides were measured using enzyme immunoassay kits (Cayman Chemical, Ann Arbor, MI, USA). The data were analyzed using a two-way analysis of variance followed by Bonferroni correction with the criterion for significance set at *P<*0.05.

### Immunofluorescent staining of mouse cerebellum

Adult (2 months old) male wild-type (C57Bl6/J) and *mdx*^*5cv*^ aged-matched male mice were perfusion fixed with 4% paraformaldehyde (Electron Microscopy Sciences, Hatfield, PA, USA). Each mouse cerebellum was then placed in a 30% sucrose/1 × phosphate-buffered saline solution overnight. Fifteen micron sagittal brain cross-sections from wild-type and mdx5cv mice were rinsed in phosphate-buffered saline and blocked in 5% normal goat serum (Sigma-Aldrich, St. Louis, MO, USA, catalog G9023) 0.1% Triton X-100 for 2 h at room temperature. A calbindin mouse monoclonal antibody (Sigma-Aldrich, C9848) and dystrophin antibody (6-10; rabbit polyclonal previously generated by our laboratory^3^) were applied in blocking solution at 1:500 and 1:100 dilutions, respectively, overnight at 4 °C. Secondary antibodies (Life Technologies, Carlsbad, CA, USA; catalog A11019, A11008) raised against rabbit and mouse IgG that were conjugated to fluorochromes were applied in blocking solution at 1:500 for 2 h at room temperature. The slides were mounted with Vectashield with DAPI (Vector Laboratories, Burlingame, CA, USA, H-1200). The images were acquired using a Nikon II microscope (Nikon Instruments, Melville, NY, USA) using OpenLab software version 3.1.5 (Improvision/PerkinElmer, Waltham, MA, USA). The images were later modified using Adobe Photoshop CC (Adobe Systems; 2015 version, San Jose, CA, USA).

### Statistics

Unless otherwise stated, one- or two-way analysis of variance (with Bonferroni correction) was used to determine significance (*P*-values) among each of the cohorts. The calculations of significance and statistical tests were done using freely available GraphPad software (www.graphpad.com; GraphPad Software, La Jolla, CA, USA). For all neurobehavioral experiments, the experimenter was double-blinded to the drug compound (or vehicle control) given and to the genotypes of the cages of mice. The double-blind experiments were decoded following completion and independent analysis of the data in cooperation with the Boston Children's Hospital IDDRCH Neurodevelopment Behavioral Core (NBC) staff.

## Results

**Figure 1:**
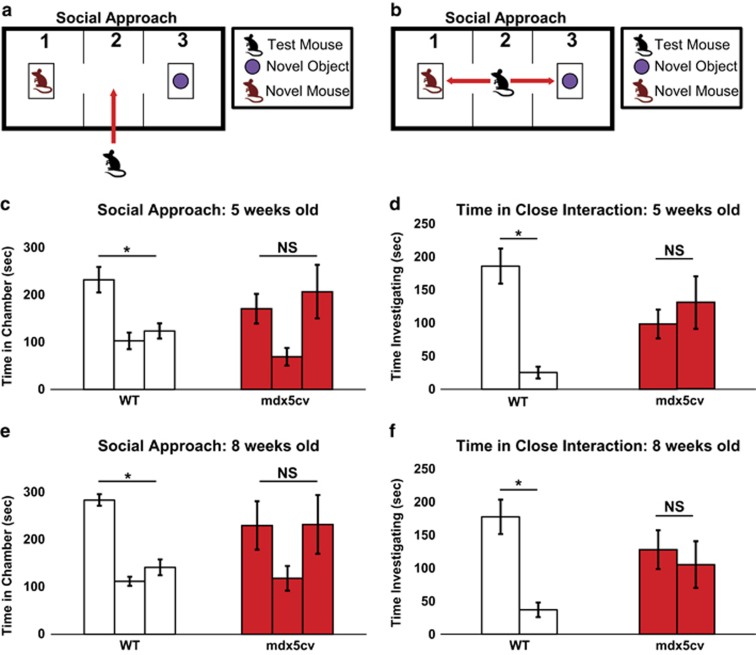
Dystrophic mice have significant neurobehavioral social deficits from juvenile to adult stages. (**a** and **b**) Schematic showing the layout of the social approach experiment in which the mouse is placed in the center chamber (2) and will move towards the novel mouse or novel object. (**c**–**f**) Social approach data at 5-week-old (**c** and **d**) and 8-week-old (**e** and **f**) wild-type (WT; open bars) and *mdx*^*5cv*^ (red bars) male mice show persistent neurobehavioral deficits in the dystrophin mice when compared with age-matched wild-type controls. The social approach F-value of time spent in each chamber for WT mice at 5 weeks of age was F_1,8_=12.834 and at 8 weeks of age was F_1,8_=23.491. The social approach F-value of time spent in each chamber for *mdx*^*5cv*^ mice at 5 weeks of age was F_1,8_=1.398 and at 8 weeks of age was F_1,8_=1.09. (**c** and **e**) The left bar in the figure represents chamber 1 (novel mouse), the middle bar chamber 2 (center point) and right bar chamber 3 (novel object). (**d** and **f**) The left bar in the figure represents chamber 1 (familiar mouse) and the right bar chamber 3 (novel object). The time investigating F-value for WT mice at 5 weeks of age was F_1,8_=20.134 and 8 weeks of age was F_1,8_=39.175. The time investigating F-value for *mdx*^*5cv*^ mice at 5 weeks of age was F_1,8_=4.44 and 8 weeks of age was F_1,8_=3.54. For all the panels, *P-value <0.05 and NS, no significance. Eight mice per cohort were used for each social approach experiment. We used a 95% confidence interval for significance (*P<*0.05) with eight degrees of freedom.

We tested normal (wild-type) and *mdx*^*5cv*^ (dystrophin exon 10 mutation^52^) male mice at 5 weeks of age (juvenile stage of development) to determine whether there were any neurobehavioral deficits associated with dystrophin deficiency. We used the three-chambered assay to measure the social approach behavior of the test mouse as previously described^50^ (Figures 1a and b). The wild-type mice spent more time interacting with the novel mouse compared with the novel object. Conversely, the *mdx*^*5cv*^ mice spent equal time interacting with the novel object and novel mouse (Figures 1c and d). These differences were still evident in a repeated testing of the same cohorts of mice at the 8-week time point (young adult), where again the *mdx*^*5cv*^ mice showed the same apparent lack of preference for the novel mouse over the novel object (Figures 1e and f). We observed similar social approach deficits in 8-week-old *mdx* mutant mice (Supplementary Figure 2), confirming this phenotype in two different dystrophin-deficient mouse lines. Thus, neurobehavioral social behavioral deficits are present in juvenile dystrophic mice and persist into adulthood, suggesting that these mice may provide a suitable preclinical model for testing pharmacological therapeutic interventions that would treat ASD social behavior symptoms associated with DMD. In addition, we observed muscle performance (increased treadmill running time to exhaustion) and overall functional improvement in the *mdx*^*5cv*^ mice given the PDE5A inhibitor as previously described in a similar study involving *mdx* mice^36^ (data not shown); however, no significant improvement in muscle performance was observed in the *mdx*^*5cv*^ mice treated with the PDE9A inhibitor.

Purkinje cells act as the primary projection neuron from the cerebellum to carry integrated sensory information back to the frontal cortex by way of the ventral tegmental area or thalamus.^53^ Electrophysiological analysis of Purkinje neurons from 3-month-old dystrophin-deficient *mdx* mice show reduced postsynaptic long-term depression.^54^ To better understand the relationship between reports of defective long-term depression and cGMP signaling in the cerebellum of dystrophin-deficient mice, we measured the cGMP content across the brain regions of *mdx* mice (Table 1). We found that although cGMP levels across most brain regions were not significantly affected by the genotype of the animals, the cerebellum of *mdx* mice displayed a pronounced deficit in cGMP production as compared with wild-type animals (Table 1). We performed a similar analysis on the WT (C57BL/6 J strain) and *mdx*^*5cv*^ (C57BL/6 J strain) backgrounds, and observed a similar decrease in cerebellum region of *mdx*^*5cv*^ mice (Table 1). These findings suggest that basal cerebellar cGMP production is dependent upon normal dystrophin production to function in a physiological range.

**Figure 2:**
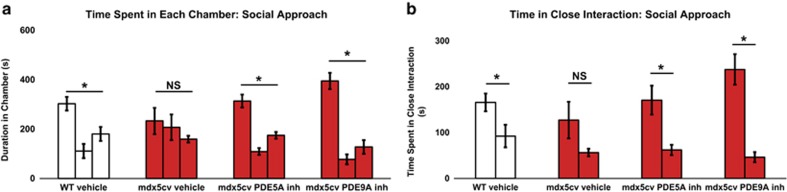
Treatment of dystrophic mice with PDE inhibitors rescues neurobehavioral social deficits. (**a** and **b**) Social approach data for wild-type (WT; open bars) and dystrophic (*mdx*^*5cv*^; red bars) mice given either vehicle, PDE5A inhibitor (sildenafil citrate; 400 mg ml^−1^) or the PDE9A inhibitor (200 mg ml^−1^). (**a**) The left bar in the figure represents chamber 1 (familiar mouse), the middle bar chamber 2 (center point) and right bar chamber 3 (novel object). (**b**) The left bar in the figure represents chamber 1 (familiar mouse) and the right bar chamber 3 (novel object). The data show that the *mdx*^*5cv*^ mice show no preference for the novel mouse or object in the vehicle-treated mice; *P-value <0.05; NS, no significance. Eight mice per cohort were used for each social approach experiment. The time spent in each chamber F-value for WT vehicle mice was F_1,8_=6.04 and the time in close interaction F-value was F_1,8_=4.82. The time spent in each chamber F-value for *mdx*^*5cv*^ vehicle-treated mice was F_1,8_=0.63 and the time in close interaction F-value was F_1,8_=0.89. The time spent in each chamber F-value for *mdx*^*5cv*^ PDE5A inhibitor-treated mice was F_1,8_=20.10 and the time in close interaction F-value was F_1,8_=9.69. The time in close interaction F-value for *mdx*^*5cv*^ PDE9A inhibitor-treated mice was F_1,8_=23.93 and the time in close interaction F-value was F_1,8_=50.10. A 95% confidence interval for significance (*P<*0.05) with eight degrees of freedom was used.

We examined whether the social behavior deficits in the *mdx*^*5cv*^ mice could be rescued via treatment with selective inhibitors of the cGMP-specific phosphodiesterases PDE5A and PDE9A, which are both highly concentrated in the cerebellar Purkinje cells.^44^ We selected a previously characterized selective and brain penetrant PDE9A inhibitor^46^ and the PDE5A inhibitor sildenafil^55^ for preclinical therapeutic trials in the *mdx*^*5cv*^ mouse model. Sildenafil was administered to wild-type or *mdx*^*5cv*^ mice at 400 mg ml^−1^ via *ad libitum* inclusion in the drinking water, and the PDE9A inhibitor was included at 200 mg l^−1^ starting at 4 weeks after birth for eight continuous weeks and was adapted from a previously described dosing regimen.^47^ Significantly, treatment of the *mdx*^*5cv*^ mice with either cGMP-specific PDE inhibitor demonstrated rescue of the social approach deficits following 1 month of treatment in all drug concentration groups. (Figures 2a and b). Furthermore, we observed no significant hindrance in any of the mouse, drug, or strain cohorts in their ability to move between chambers (Supplementary Figure 4), suggesting that any muscular deficits resulting from dystrophin deficiency did not impact on the social neurobehavioral findings between normal and *mdx*^*5cv*^ mouse strains.

The social novelty paradigm represents another three-chamber neurobehavioral assay in which the test mouse must chose to enter a chamber containing either a familiar mouse or a novel mouse (Supplementary Figures 3A and B). The wild-type and *mdx*^*5cv*^ vehicle-treated control cohorts of mice showed an expected slight preference for the novel mouse over the familiar mouse (Supplementary Figures 3C and D). Interestingly, this preference shifted towards no preference for either mouse in the PDE inhibitor-treated *mdx*^*5cv*^ mouse cohorts (Supplementary Figures 3C and D). It is unclear why the PDE inhibitor-treated *mdx*^*5cv*^ mouse cohorts showed a shift in mouse preference; however, given that the *mdx*^*5cv*^ mice only slowed a slight preference for the novel mouse, it is possible that the social novelty experiment is not robust enough to detect neurobehavioral deficits in dystrophin-deficient mice. Indeed, the C57BL6/J strain (same strain as the *mdx*^*5cv*^ mice used) has demonstrated significant variability and failed mouse preference in the social novelty assay on this strain background.^56^

## Discussion

The aggregate results from our study imply that treatment of neurobehavioral symptoms associated with developmental disturbances stemming from dystrophin deficiency and impaired cGMP signaling in the cerebellum may be improved by using either a PDE9A specific inhibitor or a PDE5A inhibitor. This therapeutic strategy may provide a potential benefit for human patients with symptoms on the autism spectrum. Ongoing clinical trials (ClinicalTrials.gov Identifier: NCT01865084; Note: while this manuscript was in revision, this trial was halted in Phase 3 owing to a failure to meet therapeutically defined end points) will test the effect of the PDE5A inhibitor tadalafil on muscle function and muscle oxygenation in DMD boys. Future trials for PDE5A and PDE9A in DMD may consider selecting subsets of DMD patients with ASD symptoms for treatment during critical periods of cerebellar circuit refinement with the inclusion of standard measures of cerebellar circuit function, such as conditioned eyeblink, as well as social behavior. Although PDE inhibitors show efficacy in the amelioration of skeletal muscle pathologies in DMD animal models and patients, it is important to note that PDE inhibitors showed no therapeutic benefits in improving cardiomyopathies in DMD and BMD patients.^57^ It is possible that the beneficial effects observed in DMD and BMD patients treated with PDE inhibitors may be resulting from the increased blood flow in their skeletal muscles.^58^ Patients with significant arterial blockages, who were treated with sildenafil, showed significant increases in NO-mediated signaling and increased oxygenation in their skeletal muscles with vasculature remodeling.^59^ The effects of PDE inhibitor administration on the vasculature system in DMD and BMD boys is further warranted.

During the submission of our manuscript, two additional studies also identified neurobehavioral social deficits in *mdx* mice.^60, 61^ Although there were some variations in our findings on the severity of these defects, these may be attributed to differences in methods used to evaluate neurobehavior and age of examined mice used in each study. Our current findings enhance previous findings that PDE9A inhibitors elevate cGMP^62^ and enhance synaptic plasticity and cognitive function in rodents.^62, 63, 64, 65, 66^ PDE9A inhibitors are currently being pursued for the treatment of mild cognitive impairment and early-stage Alzheimer's disease by several pharmaceutical companies. Our data suggest that PDE9A inhibitors may additionally be considered for treating DMD-associated autism as well as other cerebellum defined subsets of ASD. These findings pave the way to evaluate these therapeutic approaches in other subsets of ASD with developmental cerebellar dysfunction.

## Figures and Tables

**Table 1:** Dystrophic mice have reduced cGMP levels in their cerebellum

*Cohort*	*Cerebellum*	*Striatum*	*Hippocampus*	*Frontal cortex*
*(A) Mean (pmol mg^−1^ of tissue±s.e.m.)*				
WT (mixed)	0.709±0.063	0.080±0.008	0.136±0.007	0.150±0.011
* mdx*	0.392±0.033*	0.100±0.006	0.107±0.004	0.130±0.009
WT (C57BL/6J)	0.398±0.097	0.123±0.020	0.104±0.008	0.173±0.032
* mdx5cv*	0.200±0.036*	0.114±0.014	0.092±0.006	0.159±0.024
				
*(B) Average % normalized*				
WT (mixed)	100	100	100	100
* mdx*	55	126	79	87
WT (C57BL/6J)	100	100	100	100
* mdx5cv*	50	93	89	92

*P<0.05.

(A) Mean amount of cGMP levels in four regions of the brain (cerebellum, striatum, hippocampus and frontal cortex) in wild-type (WT; mixed C57BL/10ScSn strain) and *mdx* (mixed C57BL/10ScSn strain) mice. A second comparison of mean cGMP levels in the same four brain regions was performed between WT (C57BL/6 J) and *mdx*^*5cv*^ (C57BL/6 J strain). The cGMP levels are measured as pmol per mg of tissue with ±s.e.m. shown next to the cGMP value. (B) Average percent (%) values (normalized to WT at 100%) of *mdx* mice cGMP levels. Seven mice were used (*N*=8) from each cohort, and a Student's *t*-test (paired, two-tailed) was used to determine the significance between the WT (mixed) and *mdx* cohorts.
