# Supplementary material for: Reversal of neurobehavioral social deficits in dystrophic mice using inhibitors of phosphodiesterases PDE5A and PDE9A
Source: Transl Psychiatry. 2016 Sep 27;6(9):e901–. doi: 10.1038/tp.2016.174 (PMC5048211; doi:10.1038/tp.2016.174)
Supplement: Supplementary Figure 2 [file tp2016174x2.pdf]

Supplemental Figure 2

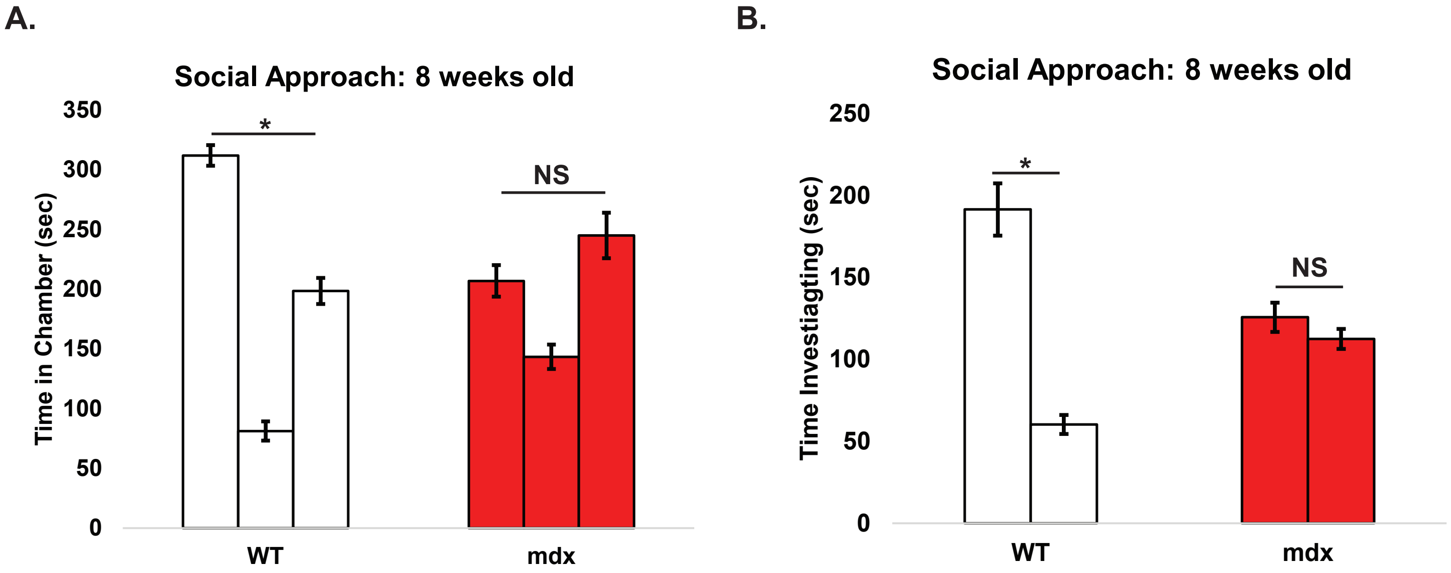

Supplemental Figure 2. The *mdx* mutant mice show social approach neurobehavioral deficits compared to wild type mice.
