# Supplementary material for: Reversal of neurobehavioral social deficits in dystrophic mice using inhibitors of phosphodiesterases PDE5A and PDE9A
Source: Transl Psychiatry. 2016 Sep 27;6(9):e901–. doi: 10.1038/tp.2016.174 (PMC5048211; doi:10.1038/tp.2016.174)
Supplement: Supplementary Figure 3 [file tp2016174x3.pdf]

Supplemental Figure 3

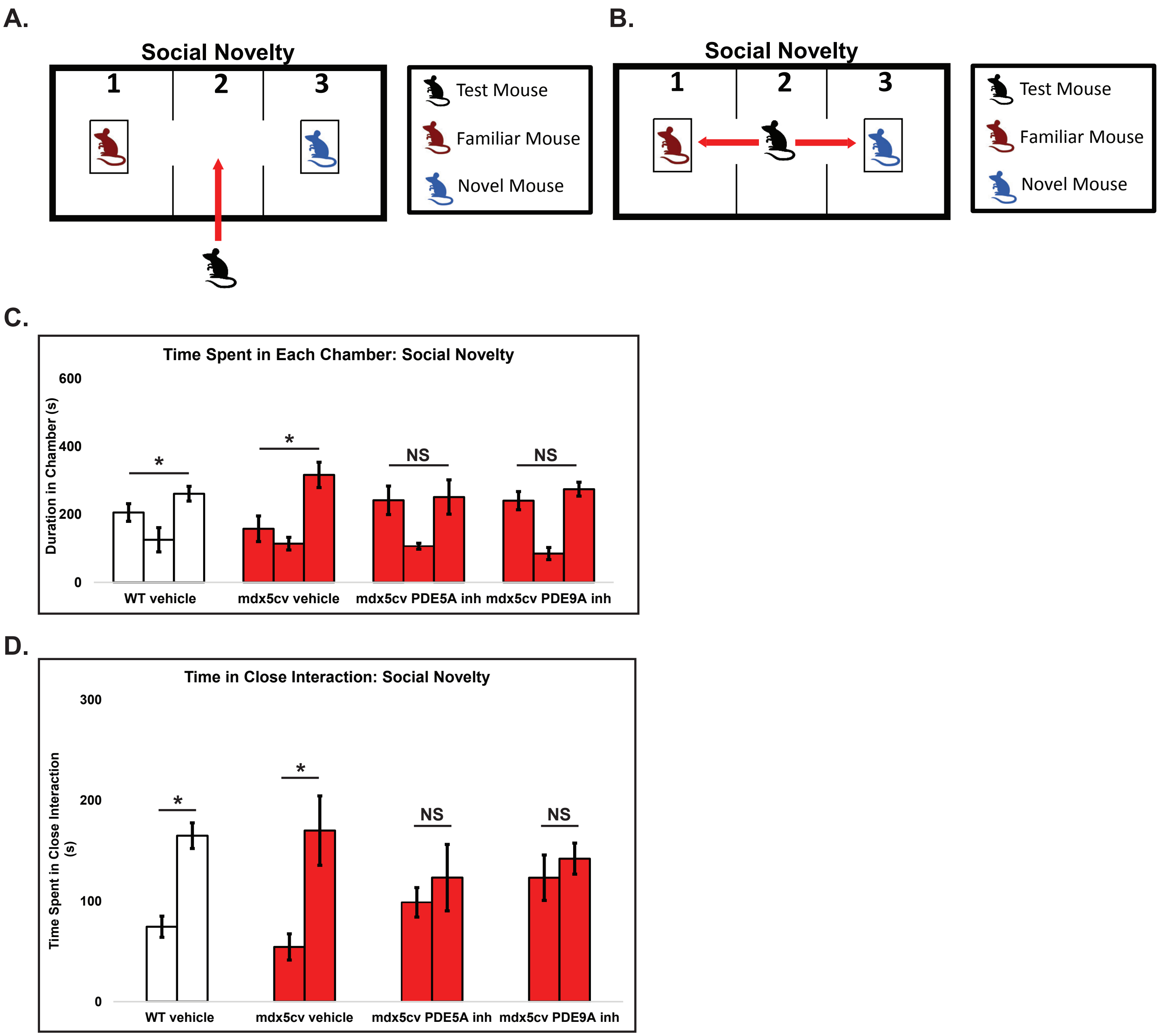

Supplemental Figure 3. Social novelty data in PDE inhibitor-treated adult wild type and mdx5cv mouse cohorts.
